# Conservation of the Dental Pulp

**Published:** 1888-04

**Authors:** J. R. Callahan

**Affiliations:** Hillsboro, O.


### Conservation of the Dental Pulp.

BY J. R. CALLAHAN, D.D.S., HILLSBORO, O.

[Read before the Mississippi Valley Dental Association, held at Cincinnati, O., March 7, 1888.]

For ages the dental pulp has been the subject of much speculation. At the present day, at almost every dental society meeting, more or less is said of the treatment of this very delicate organ. In almost every dental journal of the day, more or less is said of the protection or destruction of the dental pulp. Oftentimes the speakers or writers, as the case may be, are so positive of the supremacy of their methods or theories that they will listen to no arguments contrary to their expressed opinions. At one time the conservation, or the saving or protecting of the dental pulp, seemed to be the only recognized practice, and it was at the risk of his professional reputation that any dentist dare intimate that he was not able to preserve alive and in perfect health, for an indefinite length of time, every scrap of nerve tissue that might remain in any tooth that was placed under his professional care. Of late years a reaction seems to have set in; until to-day it seems, in some quarters, we have reached the opposite extreme. At the last meeting of the Ohio State Dental Society, the devitalization theory appeared to reign supreme. Let us hope we have done with extremes and may each and all of us find our level on this very important point.

It is the object of this paper, 1st, to provoke discussion; 2nd, to direct your attention to a plan of procedure that has proven more satisfactory to the writer than any method yet tried. Please to note we do not claim perfection, to be perfect we should be able to attain the excellancy claimed by some of the enthusiastic pulp cappers of 1886–7. Not having reached the dizzy heights of those days, let us compare notes and see what we have accomplished. To save going into tiresome details and to make our position somewhat clear, I will say at once that in practice I do not attempt to cap more than *one* exposed pulp in *ten*, believing that the arsenic should be applied to all pulps that cannot be restored to perfect health. It is my desire to call your attention more to the treatment and filling of deep seated caries or near exposures of the pulp, if you please. In a large per-cent of the mouths presented for treatment, you find the decay has penetrated the tooth to such a depth that, if all the decayed dentine was removed the pulp would be exposed. In fact, it is generally the case that the *pain* in *this* class of cavities is what has sent the person to the dentist. The teeth most likely have been giving trouble for some time. Such a case, as you all know, needs careful and intelligent treatment. We should bear in mind that we have to deal with a very delicate organ confined in a narrow, bony chamber; an organ, that while it is devoid of tactile sense, will not bear compression in the slightest degree, and is *extremely* sensitive to thermal changes. Having these most important facts in mind we may proceed to the treatment of these deep seated caries. Great care should be exercised to prevent uncovering the pulp, being careful at the same time to not leave too great a thickness of decay over the pulp. The debris should always be removed by a gentle stream of warm water. After preparing the cavity and applying the rubber dam, the layer of partially decayed dentine over the pulp should be throughly sterilized, using for this purpose 95 per cent. carbolic acid, having the acid about the same temperature of the tooth; dry the cavity thoroughly, using alchohol and warm air, then cover the decayed dentine with a thick paste of iodoform and carbolic acid, or oil of cloves if you prefer, over this flow a thin paste of oxyphosphate of zinc, and give this plenty of time to harden, when the filling may be completed with cement or gutta-percha. These fillings should be allowed to remain in the tooth until you are satisfied that there is no irritation or inflammation in the pulp tissue. I have treated in this manner teeth that had been aching two or three days prior to and at the time of treatment, with most happy results. But a few days since I opened, for examination, a sixth year molar that had been filled in this manner for two years. The tooth was aching slightly at the time it was filled; upon removal of the cement filling found the iodoform dry and apparently full strength, the tooth had been perfectly comfortable all the while. The nerve, as far as I could determine, was in perfect health.

I follow almost the same treatment for capping *exposed* pulps. That is, all pulps that I believe to be in good condition. You will observe that I here pass a very important point, viz., the restoration to health of diseased pulp tissue. A case in practice will perhaps be of interest. Miss D. presented herself for treatment complaining of pain in the right superior cuspid. Upon examination found a large cavity on distal surface of that tooth, found also a small exposure of the pulp. The decay was carefully removed, a paste of iodoform and carbonic acid placed upon the exposure, a metal cap was placed over this to prevent pressure, and in this case the cavity was filled with cotton and sandarach varnish. All pain disappeared in a few minutes after the application was made. The tooth was allowed to remain in this condition for a week, when the lady returned. Found upon examination the pulp, to all appearances, to be free from irritation; rubber dam was applied, tooth thoroughly dried, paste of iodoform and carbolic acid placed over the pulp and a thin paste of oxyphosphate of zinc flowed over this. After this had hardened the cavity was filled with cement to remain two or three months, when, if there has been no unfavorable symptoms, the tooth will be filled with gold, leaving, of course, the cap as it is at present.

I recently placed a gold filling in a right superior lateral incisor in which the pulp was capped as above described. On March 17th, 1882, I found this pulp after six years trial, to be in perfect health.

As to the medical properties and action of iodoform, Gorgas tells us that it has no irritant action, and in small doses is tonic, stimulant, anodyne, alterative and disinfectant; also antiseptic and has great influence upon the nervous system. It is not necessary, in this connection, to mention its toxic effects, on account of the small doses that must be used for want of space in cavities if for no other reason. He tells us also, that when the surface of an ulcer or wound is covered with a layer of iodoform in crystals, a certain degree of absorption of the fluids secreted takes place. The products of secretion penetrating these interstices between the minute crystals of iodoform soon lose the liquid form and produce with them an impermiable crust, and under this crust cicatrization soon occurs without any retraction of the tissue.

